# Detection of branch retinal artery occlusions in Susac’s syndrome

**DOI:** 10.1186/1756-0500-7-56

**Published:** 2014-01-21

**Authors:** Stavroula Boukouvala, Saiju Jacob, Mark Lane, Alastair K Denniston, Michael A Burdon

**Affiliations:** 1 University Hospital Birmingham NHS Foundation Trust, Queen Elizabeth Hospital Birmingham, Mindelsohn Way, Edgbaston, Birmingham B15 2WB, UK; 2 Centre for Translational Inflammation Research, Division of Immunity and Infection, University of Birmingham, Birmingham B15 2WB, UK

**Keywords:** Susac’s syndrome (SS), Retinopathy, Fluorescein angiography

## Abstract

**Background:**

We report an interesting case of asymptomatic retinal involvement in an encephalopathic patient enabling early identification of Susac’s syndrome.

**Case presentation:**

A 39-year-old Caucasian lady with hearing loss and encephalopathy was referred for ophthalmic assessment, including screening for branch retinal artery occlusions characteristic of Susac’s syndrome. Clinical features included severe headaches, right-sided hypoacusis, dysphasia and poor memory. Routine blood tests were normal. MRI brain showed numerous hyperintense lesions mainly in corpus callosum. Although she was visually asymptomatic, dilated funduscopy detected bilateral multiple peripheral branch retinal artery occlusions which were confirmed on fluorescein angiography. She was subsequently started on intravenous steroids and pulsed cyclophosphamide which improved her symptoms within 48 hours. Full recovery was made with no new arterial occlusions on four months follow-up.

**Conclusion:**

The case further establishes the crucial role of a detailed ophthalmic examination supported by fluorescein angiography in the assessment of these patients, who are at risk of being misdiagnosed and undertreated.

## Background

Sucas’s syndrome (SS) is a retinocochleocerebral vasculopathy. It is characterized by the typical triad of encephalopathy, retinopathy and sensorineural hearing loss, which was first described by J.O Susac in 1979 and since when around 300 cases have been reported [1-3]. The vast majority of them have been published within the last two decades which changed the initial perception about the rare prevalence of the disease.

SS manifests more often in women between their third and fourth decade [2]. The pathogenesis of this microangiopathy remains questionable. It is likely that autoimmune dysfunction plays a crucial role as anti-endothelial cell antibodies have been detected in studies where analysis of serum sample from patients with SS was made [4,5]. However, further research is required in order to explain the aetiology behind this autoimmune process which leads to the damage of peripheral vessels of specific organs in SS. It is of interest to note that histological examinations in specimens from a patient with SS suggest that the cause of the retinal arterial wall plaque is the accumulation of serous deposits between the retinal blood vessels and the internal limiting membrane which leads to compression or even occlusion of them. In their report McLeod *et al.* noted that capillaries with thickened walls and narrow lumens around the optic nerve head were surrounded by corpora amylacea formations which they suggested were likely to be the result of microinfarcts arising from a capillary angiopathy [6].

It has been suggested that retinopathy with branch retinal artery occlusions (BRAO) and hearing loss are not always essential in order to have the diagnosis of SS as long as encephalopathy and pathognomonic radiological findings are present [2]. Multiple sclerosis (MS) or acute disseminated encephalomyelitis (ADEM) can mimic SS not only in symptoms but also in MRI changes, which results in a late diagnosis or even misdiagnosis of SS. This is unfortunate as SS can often be treated successfully, with no recurrence [7].

Careful interpretation of radiographic findings may establish the correct diagnosis. In SS the corpus callosum is affected with multifocal lesions of size 3–7 mm, centrally located and with a ‘punched-out’ appearance. Leptomeningeal enhancement may also be positive but is not always present. Lesions in patients with MS and ADEM typically involve the undersurface of the corpus callosum and leptomemingeal involvement is not typical [8].

Due to its underlying immunopathogenesis, SS is being widely treated with high dose intravenous corticosteroids plus a second immunosuppressant. Although the rarity of the disease means that there are no randomized controlled treatment trials, the literature provides reports of cases and case series treated with agents such as intravenous immunoglobulin, mycophenolate mofetil, cyclophosphamide, plasma exchange, azathioprine, rituximab and methotrexate have been used [3,9,10]. Furthermore long-term treatment with Aspirin may be added to the immunosuppressive treatment in cases where BRAO is confirmed [11]. Li *et al.* recommend hyperbaric oxygen to attempt to reverse visual loss, although this is not generally practiced [12]. It would appear that early and aggressive treatment is associated with a steady recovery of symptoms with an excellent prognosis.

We present here a case of a 39-year-old female who presented acutely to the neurology team with rapidly progressive encephalopathy and hearing loss. The possibility of SS was raised following investigation with brain imaging which showed suggestive corpus callosal lesions. A referral to the Ophthalmology department was made and the diagnosis was confirmed by the presence of multiple branch retinal artery occlusions.

## Case presentation

**Figure 1:**
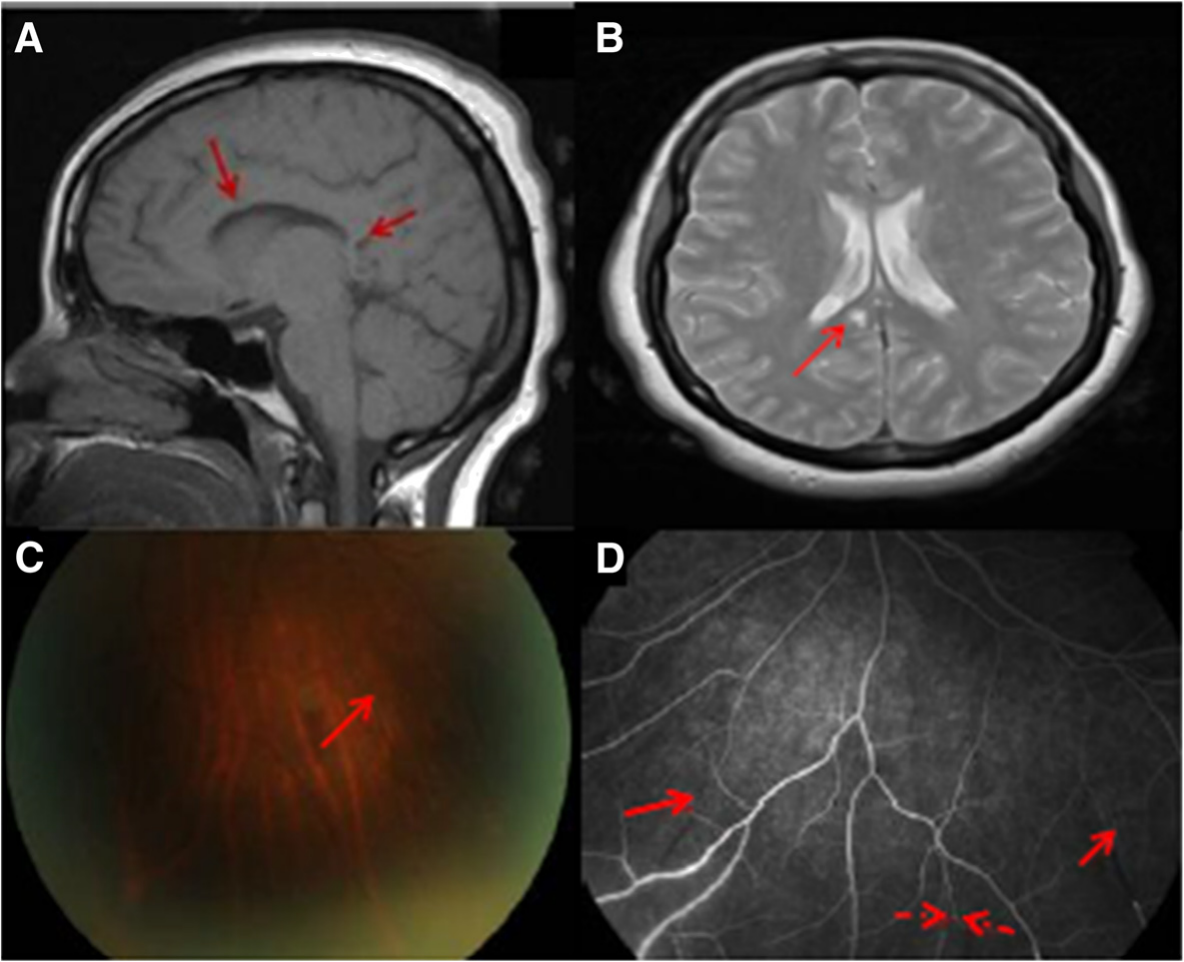
**SS findings on MRI brain (A, B), fundus photographs (C) and fundus angiography (D). A**. T1-weighted MRI brain showing punched out lesions in the corpus callosum (arrowed) typical of Susac’s syndrome. **B**. Axial T2- weighted MRI brain showing typical “snow ball” appearance of inflammation in the posterior corpus callosum (arrow) **C**. Colour fundus photograph showing branch retinal artery occlusion (arrowed) in the peripheral fundus (OS). **D**. Fundus fluorescein angiographic image showing the same lesion (broken arrow) and additional occlusions not previously recognised on clinical examination alone (unbroken arrows).

A 39-year-old Caucasian lady presented with five weeks of severe vertigo, headaches and rapidly progressive confusion, following a trip to Tunisia. She reported no visual symptoms. She had a past medical history of bipolar disorder diagnosed two years previously. Neurological examination revealed right-sided hypoacusis, dysphasia, ataxia and poor memory. Haematological and biochemical profile was normal. Serological screening for infectious agents was negative. Lumbar puncture revealed marginally elevated opening pressure, raised protein with no cells and no oligoclonal bands. A T1-weighted brain MRI revealed inflammatory changes, with multiple ‘punched-out’ lesions in the corpus callosum (Figure 1A). On T2- weighted MRI “snow ball” appearance of inflammation in the posterior corpus callosum was found (Figure 1B). These features were identified as being suggestive of SS, and thus an ophthalmology opinion was sought. On ophthalmic examination, patient’s best corrected visual acuity was 6/9 in the right and 6/6 in the left eye. There was no afferent pupillary defect or abnormal colour vision in either eye. Dilated fundal examination revealed healthy discs and maculae but multiple BRAO bilaterally (Figure 1C), which were found to be even more widespread on fluorescein angiography (Figure 1D). The rest of ophthalmic examination was otherwise unremarkable. A diagnosis of SS was made. Treatment with intravenous methylprednisolone and cyclophosphamide resulted in stabilisation of disease within the first 48 hours. The patient made a steady recovery of function, including mobility, speech, hearing and memory. On six weeks ophthalmology follow-up she had an unaided vision of 6/5 bilaterally without new arterial occlusions. No relapses have been noted after a period of ten months.

## Conclusions

The diagnosis of SS is challenging. The classic triad of SS may not be present at the onset of the disease and the retinal involvement which is commonly asymptomatic may be missed.

Although headache is the commonest initial symptom for any manifestations of encephalopathy, transient personality and mental changes are also common in SS [13]. Interestingly psychiatric symptoms mimicking bipolar spectrum disorder have been described in a case of SS in the past but it was unclear whether they were truly related to SS as they started three decades after the initial SS clinical signs [14]. Our patient suffered from bipolar disorder diagnosed two years previously, but we do not think that this has any relation to her Susac’s syndrome.

SS-associated retinal artery occlusions are of importance both because they may cause severe visual loss (depending on the arteriole involved) but also because they may hold the key to rapid diagnosis and early initiation of immunosuppression. In SS, retinal artery occlusions have a characteristic pattern on fluorescein angiography with arterial wall hyperfluorescence due to atheromatous plaques, also called “Gass plaques” and are usually distally located from the bifurcation [15]. Indocyanine green angiography shows normal choroidal circulation [16]. Optical coherence tomography (OCT) may also be useful in the clinical challenge of differentiating SS from MS. Brandt *et al.* have reported that Susac patients show a more distinct sectorial pattern of reduction in both macular and retinal nerve fibre layer thickness in contrast to the more diffuse changes seen in MS patients [17]. Seven-Tesla MRI may also aid in further refinement in terms of identifying the characteristic radiological changes observed in SS. In their study of five patients with SS and ten age and sex-matched patients with MS, Wuerfel *et al.* observed that using the T2-weighted FLASH (Fast Low Angle SHot) sequences, most (92%) white matter lesions in MS were centred on a small vein and were often (41%) characterized by a hypointense rim, whereas white matter lesions in SS were less commonly (54%) surrounding an identifiable vessel and rarely (4%) exhibited a hypointense rim. The authors also noted callosal atrophy and CSF-isointense lesions within the central part of the corpus callosum [18].

Through this case we want to make physicians more aware of the possibility of visually asymptomatic patients with retinal involvement in SS and suggest detailed fundoscopic and angiographic assessment by a neuro-ophthalmologist or retinal specialist in cases where neurological and radiological investigations raise the possibility of SS.

## Consent

Written informed consent was obtained from the patient for publication of this case report and any accompanying images. A copy of the written consent is available for review by the Editor of this journal.

## Abbreviations

SS: Sucas’s syndrome; MS: Multiple sclerosis; ADEM: Acute disseminated encephalomyelitis; BRAO: Branch retinal artery occlusion; OCT: Optical coherence tomography; FLASH: Fast low angle shot.

## Competing interests

The authors declare that they have no competing interests.

## Authors’ contributions

SB carried out information gathering, literature search, drafting of case report. SJ involved in diagnosis of condition, approved the final manuscript. ML case information gathering, literature search. AD performed tests on patient, carried out literature review, helped to draft the case, and approved the final manuscript. MAB confirmed diagnosis, proposed publication of case, and approved the final manuscript. AKD and MAB share the role of senior author. All authors have read and approved the final manuscript.

## Authors’ information

Alastair K Denniston and Michael A Burdon share the role of senior author.
